# Interval and continuous exercise enhances aerobic capacity and hemodynamic function in CHF rats

**DOI:** 10.1590/bjpt-rbf.2014.0098

**Published:** 2015-09-01

**Authors:** Ramiro B. Nunes, Jadson P. Alves, Luíza P. Kessler, André Z. Dornelles, Giuseppe P. Stefani, Pedro D. Lago

**Affiliations:** 1 Laboratório de Fisiologia, Universidade Federal de Ciências da Saúde de Porto Alegre (UFCSPA), Porto Alegre, RS, Brazil; 2 Programa de Pós Graduação em Ciências da Reabilitação, UFCSPA, Porto Alegre, RS, Brazil

**Keywords:** rehabilitation, interval training, aerobic capacity, chronic heart failure

## Abstract

**OBJECTIVE:**

The aim of the present study was to compare the effects of continuous versus interval aerobic exercise training on hemodynamic parameters, cardiac remodeling, and maximal exercise capacity (MEC) in chronic heart failure (CHF) rats.

**METHOD:**

Twenty-four male Wistar rats were subjected to myocardial infarction (MI) surgery. Five weeks post MI, the animals were assigned to one of three groups: sedentary group (CHF-Sed, n=8), aerobic continuous training group (CHF-ACT, n=8), and aerobic interval training group (CHF-AIT, n=8). Treadmill training was performed five times a week for 8 weeks (ACT: 50 min/day at 15 m/min and AIT: 40 min/day with 8 min of warm-up at 10 m/min and exercise at 15 m/min 4×4 min interspersed with 4×4 min at 23 m/min). MEC was evaluated pre and post exercise program.

**RESULTS:**

Left ventricular end-diastolic pressure (LVEDP), left ventricular mass/body mass ratio (LVM:BM), and total collagen volume fraction were lower in the trained groups compared with the sedentary group, but no difference was found between the trained groups. Systolic ventricular pressure (SVP) and maximum positive derivative of LV pressure (+dP/dt_max_) were higher in the trained groups, but CHF-ACT showed higher +dP/dt_max_ compared to CHF-AIT. Both training regimens were able to increase MEC. However, the aerobic interval training was superior for improving MEC.

**CONCLUSION:**

Aerobic training is an important intervention to improve cardiac function and remodeling and physical capacity in CHF rats. Interval training is a potential strategy to maximize the results, but exercise type and intensity are still topics to be explored.

## Introduction

Early fatigue and exercise intolerance are the main manifestation of chronic heart failure (CHF) syndrome. This reduction in physical capacity is due to abnormalities in both cardiovascular and skeletal muscle function^1^^,^ 2. The chronic inflammatory state and impaired perfusive O_2_ transport to active muscle leads to an imbalance between O_2_ delivery and requirements creating an accentuated intracellular metabolic perturbation and enhanced glycogenolysis^3^. Cardiac dysfunction is strongly associated with a progressive cardiac remodeling, which is influenced by hemodynamic overload, neurohormonal activation, and pro-inflammatory state^4^.

Exercise training is a safe non-pharmacological intervention in stable CHF patients with positive effects on both morbidity and quality of life^5^. The body of evidence confirms that aerobic exercise can enhance cardiac performance through several intrinsic mechanisms, such as improvement of myocardial energy metabolism, increase in myocardial perfusion and angiogenesis, and strengthening of cardiac contractility^6^^,^ 7. In addition, as demonstrated by Haykowsky et al.^8^, aerobic exercise training is an important intervention to ameliorate cardiac remodeling in CHF patients.

In recent years, several different types and intensities of aerobic exercise training have been investigated in patients^9^^-^ 11 and animal models^12^^-^ 14 of CHF. The effects of continuous aerobic exercise training in functional capacity, quality of life, and clinical outcomes in CHF patients are well recognized^15^^,^ 16, however the interval training model has been suggested as a novel and effective strategy because it has resulted in greater increases in exercise capacity than those achieved with continuous aerobic training^11^^,^ 17. In the CHF rat model, aerobic interval training was able to demonstrate improvement in cardiomyocyte contractility, myocardial hypertrophy attenuation, and myocardial expression of atrial natriuretic peptide reduction^18^.

We therefore hypothesized that aerobic interval training can be more effective than continuous training for improving hemodynamic function, cardiac remodeling, and physical capacity in CHF rats. To test this hypothesis, the present report was designed to compare hemodynamic parameters, myocardial collagen volume fraction and hypertrophy, and maximal exercise capacity in CHF rats submitted to two different types and intensities of aerobic exercise training.

## Method

### Animal model

A total of 24 male Wistar rats were used, weighing between 250 g and 270 g, obtained from the Animal Breeding Unit at *Universidade Federal de Ciências da Saúde de Porto Alegre* (UFCSPA), Porto Alegre, RS, Brazil. The rats were housed in an animal room at a constant temperature (22 °C) on a 12-hour light-dark cycle, receiving water and food *ad libitum* as previously described^19^. This study followed the ethical rules established by the Guide for the Care and Use of Laboratory Animals published by the National Institutes of Health (NIH publication 85-23, revised in 1996), and it was approved by the Animal Research Ethics Committee of UFCSPA (protocol no. 620/08).

### Myocardial infarction (MI)

The rats were anesthetized with xylazine (12 mg/kg, i.p.) and ketamine (90 mg/kg, i.p.) and placed under rodent respirator (Sam Way VR 15). Myocardial infarction was induced as previously described by Pfeffer et al.^20^ The heart was exposed and artery coronary ligation (ACL) was performed.

### Experimental design

Before myocardial infarction (MI), the rats were tested on the treadmill at their intrinsic running capacity, and only the runner rats were included. Five weeks post MI surgery, the animals were randomly assigned to one of three groups: sedentary group (CHF-Sed, n=8), aerobic continuous training group (CHF-ACT, n=8), and aerobic interval training group (CHF-AIT, n=8).

### Maximal exercise capacity measurements

Five weeks after MI surgery, all animals were submitted to a progressive treadmill running test to measure running capacity until fatigue. The test protocol was based on step-wise increases in treadmill speed (starting at 10 m/min and then the speed was incrementally increased 5 m/min every 3 min until exhaustion), as previously described^21^. The same procedures were performed again at the end of study.

### Aerobic exercise training protocols

Initially, all animals were submitted to an adaptation period (five days) and ran 10 to 30 min/day at 10 to 15 m/min. After the adaptation period, aerobic exercise training was performed five times a week, for 8 weeks, on a motorized treadmill. The aerobic training programs consisted of ACT for 50 min/day at 15 m/min (60% of maximal speed obtained in the test) and AIT for 40 min/day with 8 min of warm-up at 10 m/min and exercise at 15 m/min 4×4 min interspersed with 4×4 min at 23 m/min (92% of maximal speed obtained in the test).

### Hemodynamic evaluation

Two days after the last maximal capacity test, the animals were anesthetized with ketamine (90 mg/kg, i.p.) and xylazine (12 mg/kg, i.p.). A polyethylene catheter (PE-50) was inserted into the right carotid artery for arterial pressure measurements. After 5min, the catheter was placed into the left ventricle and ventricular pressures were measured for 5 min. The pressures were recorded with a pressure transducer (Strain - Gauge - Narco Biosystem Miniature Pulse Transducer RP-155, Houston, TX, USA) coupled to a pressure amplifier (Stoelting, Wood Dale, IL, USA), and the signals were digitized with a data acquisition system (CODAS - Date Acquisition System). These data were used to determine systolic blood pressure (SBP), diastolic blood pressure (DBP), heart rate (HR), left ventricular systolic pressure (LVSP), maximum positive (+dP/dt_max_) and negative (-dP/dt_max_) derivatives of left ventricular pressure, and left ventricular end-diastolic pressure (LVEDP).

### Determination of pulmonary and hepatic congestion

The animals were sacrificed by decapitation. The lungs and liver of each animal were removed, weighed, and dehydrated (at 80 °C) for 48 h and then weighed again to evaluate the water percentage.

### Determination of infarct size, cardiac hypertrophy, and collagen content

The hearts were removed and weighed. The left ventricles were weighed and the size of the infarct area was determined by planimetry^22^. Cardiac hypertrophy was evaluated by LM mass:body mass (LVW:BM) ratios. Myocardial collagen volume was determined by histology. Cryostat sections (6 µm) of the myocardial were stained with picrosirius red (PSR). Collagen measurements were obtained from digitized images (40× magnification lens) captured with a camera attached to an Olympus BX 50 microscope. Forty microscopic fields were analyzed in the myocardial non-infarcted area, and the perivascular collagen was excluded. The total collagen volume fraction was obtained through computerized image analysis software (Image Pro plus 4.5, Media Cybernetic Inc., Silver Spring, MD, USA).

### Statistical analysis

The data are presented as mean ± SD. One-way ANOVA followed by the Student-Newman-Keuls post-hoc test was used to compare groups. Two-way ANOVA followed by Bonferroni correction was used to test groups in different times and training effects. A *P*-value of less than 0.05 was considered statistically significant. The Graph Pad Prism 5 program (Graph Pad Software, San Diego, CA, USA) for Windows was used as a computational tool for the data analysis.

## Results

### Infarcted area, pulmonary and hepatic congestion, and cardiac hypertrophy

The myocardial infarcted area was similar in all groups (Table 1), suggesting an equal disease condition among the three experimental groups. However, pulmonary congestion and LVM:BM ratio were lower in the trained groups when compared with the sedentary group (Table 1). Interestingly, no difference was found between exercise types related to these variables. Hepatic congestion did not show a difference between the groups (Table 1).

**Table 1.** Myocardial infarct size, pulmonary and hepatic congestion, and cardiac hypertrophy

Parameters	CHF-Sed	CHF-ACT	CHF-AIT	*F * *(2.22* *)*	*P*
MIS (%)	41±3	40±4	39±2	0.8602	0.43
PC (%)	75±1	73±2^*^	73±1^*^	7.402	0.0037
HC (%)	71±1	70±1	71	2.451	0.11
LVM:BM (mg/g)	3.5±0.2	2.9±0.4^*^	3±0.3^*^	11.45	0.0004

Values are shown as mean±SD. MIS: myocardial infarct size; PC: pulmonary congestion; HC: hepatic congestion; LVM:BM: left ventricular mass/body mass ratio. * P<0.05 vs. CHF-Sed. One-way ANOVA followed by the Student-Newman-Keuls post-hoc test was used for statistical analysis.

### Hemodynamic variables

As shown in Table 2, resting HR was similar between groups, but systolic blood pressure and diastolic blood pressure were higher in the trained groups compared with the sedentary group (*P*<0.05). LVEDP was lower in both interval and aerobic continuous training groups than the sedentary group. The same improvement was observed in LVSP, which was higher in trained groups when compared with the sedentary group (*P*<0.05). Additionally, the positive and negative dP/dt_max_ were higher in the trained groups than in the sedentary group (*P*<0.05). Additionally, aerobic continuous training was better for improving this parameter compared with aerobic interval training (Table 2).

**Table 2.** Hemodynamic variables.

Hemodynamics	CHF-Sed	CHF-ACT	CHF-AIT	*F * *(2.22* *)*	*P*
HR (bpm)	267±69	290±61	274±57	0.2903	0.75
LVEDP (mmHg)	30±3	18±6^*^	20±7^*^	9.760	0.001
LVSP (mmHg)	83±13	105±9^*^	103±9^*^	10.82	0.0006
+dP/dt_max_ (mmHg/s)	3341±363	5938±1327^*†^	4903±926^*^	14.92	0.0001
–dP/dt_max_ (mmHg/s)	– 2263±223	– 4198±1239^*†^	– 3293±761^*^	10.41	0.0007
SBP (mmHg)	86±18	108±22^*^	105 ±15 ^*^	4.932	0.01
DBP (mmHg)	71±7	87±14^*^	85±13^*^	4.133	0.03

Values are mean±SD. HR: heart rate; LVEDP: left ventricular end-diastolic pressure; LVSP: left ventricular systolic pressure; +dP/dtmax: maximum positive derivative of LV pressure; -dP/dtmax: maximum negative derivative of LV pressure; SBP: systolic blood pressure; DBP: diastolic blood pressure. One-way ANOVA followed by the Student-Newman-Keuls post-hoc test was used for statistical analysis. *P<0.05 vs. CHF-Sed. †P<0.05 vs. CHF-AIT.

### Body mass and maximum exercise test

Body mass was not different between groups in both moments, before and after protocol; however, body mass was significantly different within each group from pre to post protocol (*P*<0.05, Table 3). There was no difference among the groups at the beginning of the study with regard to maximal exercise capacity evaluated by test duration and maximum speed (25 m/min; data not shown). The training intensity was determined by percentage of maximum speed (ACT at 60% = 15 m/min and AIT at 60% = 15 m/min and 92%= 23 m/min). Eight weeks of aerobic exercise protocols were able to increase maximal exercise capacity when compared with pre and post measurements within each trained group. However, aerobic interval training promoted a superior gain in maximal exercise capacity compared with aerobic continuous training (*P*<0.05) (Table 3).

**Table 3.** Body mass and maximum exercise test.

	CHF-Sed		CHF-ACT		CHF-AIT	
	*Pre*	*Post*	*Pre*	*Post*	*Pre*	*Post*
Body mass (g)	257±15	343±39^*^	270±19	348±32^*^	253±21	323±36^*^
Exercise test (s)	678±105	516±98^*^	671±98	948±153^*†^	677±96	1082±145^*†#^

Values are mean±SD. Two-way ANOVA followed by the Bonferroni post-hoc test was used to compare groups in pre moment and post moment. Body mass: Interaction (F(2.41)=0.3155; P>0.05), training (F(2.41)=2.189; P>0.05), time (F(2.41)=89.99; P<0.001. Exercise test: Interaction (F(2.41)=41.07; P<0.001), training (F(2.41)=38.15; P<0.001), time (F(2.41)=44.64; P<0.001. * P<0.01 from pre to post. † P<0.05 vs. CHF-Sed. # P<0.05 vs. CHF-ACT.

### Collagen content

**Figure 1.**
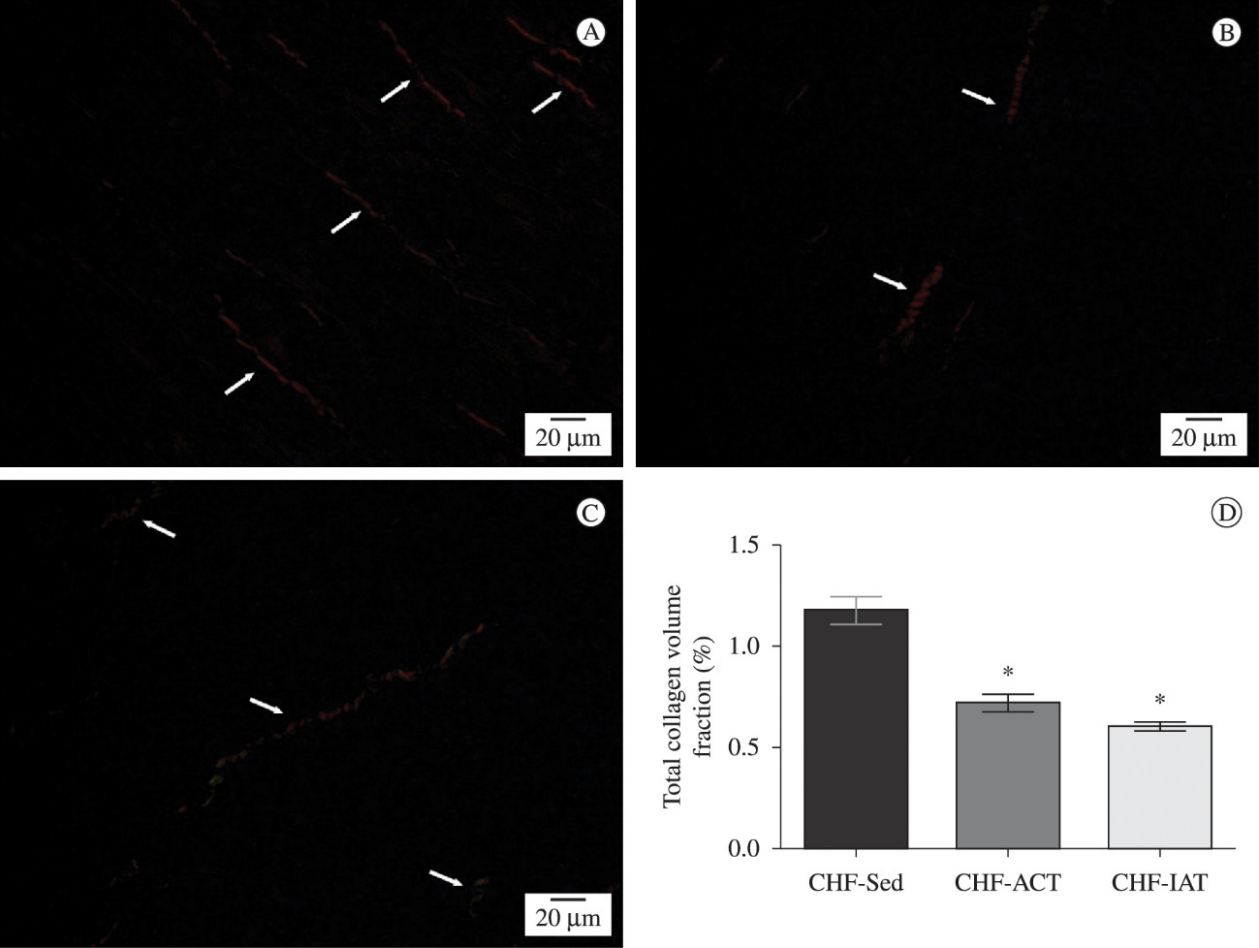
Representation of picrosirius red stained ventricular sections under polarized light. (A) CHF-Sed group; (B) CHF-ACT group; (C) CHF-AIT group; (D) Collagen mean values. Mean ± SD. F ((2,16)= 40.89; P=0.0001) * P<0.05 vs. CHF-Sed group. One-way ANOVA followed by the Student-Newman-Keuls post-hoc test was used for statistical analysis.

Figure 1A-C displays examples of picrosirius stained noninfarcted left ventricular sections under brightfield and polarized light for the three groups. In addition, Figure 1D displays statistical analysis of total collagen volume fraction. Total collagen volume fraction was significantly higher in the sedentary group than in both trained groups (CHF-Sed 1.17±0.2; CHF-ACT 0.72±0.1 and CHF-AIT 0.60±0.1%; *P*<0.05) There was no difference between the trained groups.

## Discussion

The present report is centered on three major findings that partially elucidate our hypothesis: 1) both exercise training protocols decreased LVEDP and increased PSV and positive and negative dP/dt_max_; 2) both exercise training protocols decreased LV hypertrophy and collagen volume fraction; 3) both exercise training protocols increased the maximal physical capacity, but interval training was better than continuous training.

The development of heart failure post MI is determined by the size of the necrotic area, the wound healing response, and chronic remodeling of both the infarct scar and the remote non-infarcted left ventricular myocardium^23^. The chronic neurohormonal activation, pro inflammatory state, and hemodynamic overload lead to progressive cardiac remodeling^4^ with a marked increase in collagen content and left ventricular dysfunction^24^. In the present study, collagen volume was significantly higher in the CHF-Sed than the CHF-ACT and CHF-AIT. However, there was no difference between the trained groups. This result suggests that aerobic exercise training is able to attenuate myocardial fibrosis independently of the exercise model. Xu et al.^13^ found a reduction in collagen volume fraction in infarcted rats submitted to 8 weeks of treadmill exercise training (5 days/week; 50 min per day at 15 m/min). This reduction was associated with a decrease in TIMP expression, suggested enhanced proteolytic activity, and attenuated excessive myocardial fibrosis^13^. In the present study, both types of training showed a similar reduction in collagen volume fraction in a noninfarcted left ventricle. Historically, it has been thought that the contribution of cardiomyocytes to cardiac remodeling is primarily caused by a disproportionate hypertrophy which occurs due to an increase in cell length in mechanically dysfunctional noninfarcted regions adjacent to a chronic transmural myocardial infarction^25^. In the present study, the analysis of cardiac hypertrophy indicated that aerobic exercise training was able to attenuate left ventricle hypertrophy as evidenced by a lower LVM:BM ratio in the trained groups compared to the sedentary group. In contrast, no differences were found between the groups trained with different modalities. Hemodynamic improvements were also observed in the trained groups when compared with the sedentary group. Eight weeks of aerobic exercise training were able to reduce LVEDP and increase LVSP and positive and negative dP/dt_max_, as well as normalize SBP and DBP in CHF rats. In addition, these hemodynamic improvements were accompanied by a reduction in pulmonary congestion.

In recent years, maximal aerobic exercise capacity has been considered the single best predictor of both cardiac and all-cause deaths among established cardiovascular disease patients^26,27^. Wisløff et al.^11^ evaluated the effects of two different intensities of aerobic exercise training in CHF patients. After 12 weeks of exercise training, VO_2max_ increased 46% and 14% in the aerobic interval training and moderate-continuous training groups, respectively, compared with the sedentary group. In our study, we found a similar result related to maximal exercise capacity in CHF rats, possibly due to the peripheral adaptations caused by the higher intensity of AIT. The animals were submitted to an exercise test pre and post-training period to compare the efficacy of the training protocols. Both protocols were able to increase this parameter, but the interval training group showed higher maximal exercise capacity compared with continuous training. CHF leads to cardiovascular and skeletal muscle abnormalities with marked impairment in peripheral energy metabolism with reduced cellular oxidative capacity^3^. Here, we found that AIT was able to induce an improvement in maximal exercise capacity.

The present study does have some limitations. Firstly, metabolic enzyme activity or cell mitochondrial content, which represent aerobic capacity of the peripheral muscle, was not measured. Secondly, echocardiography was not used to evaluate the changes in ventricular diameter and function. These analyses could provide more accurate data about the effects of physical training.

## Conclusions

Both interval aerobic training and continuous aerobic training were effective in improving hemodynamic function, cardiac remodeling, and maximal exercise capacity in CHF rats. However, interval training was better than continuous training for increasing maximal physical capacity. Exercise type and intensity are still topics to be explored in the management of CHF conditions.
